# Dealing with health literacy at the organisational level, French translation and adaptation of the Vienna health literate organisation self-assessment tool

**DOI:** 10.1186/s12913-019-3955-y

**Published:** 2019-03-04

**Authors:** Gilles Henrard, Marc Vanmeerbeek, Laetitia Buret, Jany Rademakers

**Affiliations:** 1 Department of General Practice/Family Medicine, University of Liège, Postal address: 13 avenue Hippocrate, Quartier Hôpital B23, 4000 Liège, Belgium; 2 Research Unit Primary Care & Health, University of Liège, Postal address: 13 avenue Hippocrate, Quartier Hôpital B23, 4000 Liège, Belgium; 3 Nivel, PO box 1568, 3500 BN Utrecht, the Netherlands; 4 Department of Family Medicine, CAPHRI, Maastricht University, PO Box 616, 6200 MD Maastricht, the Netherlands

**Keywords:** Health literacy (MeSH), Organisational culture (MeSH), Organisational innovation (MeSH), Organisational health literacy, Questionnaire, Translation

## Abstract

**Background:**

Efforts to address health literacy should favour a system-based approach with the dual aim both of fostering the material conditions and creating a work culture inside health care organisations that makes it easier for people to use information. The Vienna Health Literate Organisation (V-HLO) self-assessment tool is a German-speaking questionnaire for quality managers of health care organisations. Its objective is to provide a diagnostic of the strengths and weaknesses of the organisation in terms of health literacy. Our goal was to translate and culturally adapt this questionnaire for the French-speaking part of Belgium.

**Methods:**

We followed the Translation, Review, Adjudication, Pretesting, and Documentation (TRAPD) team model for cross-cultural translation of questionnaires. We used cognitive interviews with quality experts to pre-test the translation.

**Results:**

Cognitive interviews allowed us to improve the translation by removing certain ambiguities, providing contextual clarifications or rephrasing some items in such a way as to render them more culturally appropriate. Local experts generally judged the tool to be relevant and applicable to their context. The insight gained with regard to their cognitive process when completing the V-HLO allowed us to identify possible barriers to the adoption of the tool (such as difficulties in considering staff literacy as a relevant target for the tool, fear of overwhelming staff, a feeling that some items fell outside the scope of health literacy and lack of attention for integration of services with primary care) and could contribute to the future development of the tool.

**Conclusion:**

We translated and adapted the V-HLO self-assessment tool for French. The French version of the V-HLO will now be implemented in our local context to assess whether it can make it easier for people to deal with the complexities of health care organisations.

**Electronic supplementary material:**

The online version of this article (10.1186/s12913-019-3955-y) contains supplementary material, which is available to authorized users.

## Background

While the information relative to the health care environment can be extremely complex the ability to access, understand, appraise and apply health information to decision-making known as health literacy, is not equally shared between health care users. The European Health Literacy Survey revealed that 12% of Europeans have inadequate, while 35% have limited, health literacy [1]. A study in Belgium showed similar results, where only 58.7% of the population have sufficient health literacy [2]. People with low health literacy are less likely to use preventive care, have a greater number of hospitalisations, a higher level of emergency care use and a higher mortality rate among elderly individuals [3]. With an ageing population, a tendency to (over)medicalize health problems and increase pressure on patients in terms of self-management and active participation in their health care, sometimes by using e-applications, further increases this problem. It should be added that improving health literacy could also potentially increase health equity [4].

Health literacy can be considered sufficient when, in a particular circumstance, there is a balance between individual abilities and the complexity of the demands made by the system [5]. Health care professionals are particularly accountable for the second part of the equation. But efforts to lower barriers created by overly-complex information need to extend beyond merely promoting better communication by healthcare providers. System level changes are needed to better align health care demands with the capability of the public [6]. A wholes series of interrelated aspects, affecting both patients and professionals, can hinder the effectiveness of care and should be considered an institutional responsibility for example in order to easily find care providers on the way to and in the hospital, to have a minimum of time and sufficient comfort for shared decision making or to provide the patient with help in interpreting the bill that follows. Within health care organisation, those changes which necessarily involve management, should foster the material conditions and work culture that make it easier for people to navigate, understand and use information and services in order to take care of their health [7]. Moreover, many of those structural changes could benefit all users regardless of their literacy levels.

Though the concept of health literacy has been the subject of many publications over the last decades, few of these have addressed interventions, especially in Europe [8]. Furthermore, most of those interventions focus on communication skills targeting specific patient groups and very few adopt a global organisational perspective [9]. In this context, the American Institute of Medicine defined the “Ten Attributes of Health Literate Organizations” following a universal precaution approach. Building upon these Ten Attributes while adding explicit references to concepts of quality management and targeting patients, staff, and citizens, an Austrian team developed the “Vienna Concept of a Health Literate Health Care Organization”. Reviewing the growing number of theories, frameworks and implementation issues of organisational health literacy, Farmanova et al. highlighted the V-HLO as one of two existing frameworks with a vision of organisational health literacy as a complex phenomenon, understanding it as co-production of quality, safety and health promotion and focusing on developing organisational capacities, structures and processes to support action on health literacy [10].

In order to make the concept applicable to the practice of health care organisations, a self-assessment tool for hospitals and large health care organisations was developed [11]. The questionnaire comprises 9 standards, 22 sub-standards and 160 items (see Table 1). It is intended to be completed by a one-off multidisciplinary panel of individuals in charge of quality in the broadest sense (Quality and safety officer but also nurse or medical head of department, operational or logistics manager, director of human resources, for example) inside the organisation that performs the self-assessment. Is the hospital site available in several languages? Does public transport clearly indicate the destinations and stops to get there? Is there a clear guiding system within it? Are specialized and trained interpreters available? When referring to other providers, are patients helped to schedule appointments and is useful information transmitted? These are all items that lead to an “organisational diagnostic”, highlighting strengths and weaknesses of the organisation in terms of organisational health literacy. By raising awareness at management level on this usually sparsely invested area of quality of care, and by helping to identify preferred action track, the V-HLO can constitute a milestone for an organisation to become health literate [12]. Moreover, this one-off process is relatively “light” to achieve (a few hours for each participant of the panel), and thus complementary to more demanding external recurrent accreditation or participatory actions [13]. In a feasibility study in Austria, 9 hospitals adjudged the tool to be understandable, relevant and usable [14].

**Table 1:** The 9 standards of the Vienna Health Literate Organisations (adapted from Dietscher and Pelikan 2016)

Standards	Examples of items
1. Establish management policy and organisational structures for health literacy	- Financial resources and personnel for organisational health literacy are available. - Patient surveys include questions about the quality of communication.
2. Develop materials and services in participation with relevant stakeholders	- Documents and services for patients (such as information sheets, informed consent forms and apps) are developed and tested together with patient representatives. - Patients are involved in the training of staff.
3. Qualify staff for health-literate communication with patients	- When hiring new staff, importance is given to the health literacy and communication competencies of applicants. - Staff is trained with regard to collaboration with interpreters.
4. Provide a supportive environment – health-literate navigation and access	- The website is available in different languages. - The organisation can be reached by telephone 24 h a day.
5. Apply health literacy principles in routine communication with patients	- Patients are encouraged to ask questions. - Written materials are used to reinforce spoken communication and not as a substitute.
6. Improve health literacy of patients and relatives beyond hospital stay	- The organisation explicitly informs patients about self-help groups and similar support offers.
7. Improve the health literacy of staff	- The organisation continuously provides training in managing occupational health and safety risks.
8. Contribute to health literacy in the region	- Patients are supported in scheduling their post-discharge appointments with other services.
9. Share experiences and be a role model	- The organisation participates in health literacy research and development projects.

In the light of those results, a specific working group has emerged inside the International Network for Health Promoting Hospitals and Health Care Services,^1^ of which the first authors are members, with the aim of stimulating the adaptation, translation and dissemination of the V-HLO concept and self-assessment tool. The goal of this study was to perform a cross-cultural translation of the V-HLO tool adapted for the French-speaking area of Belgium.

## Methods

**Fig. 1:**
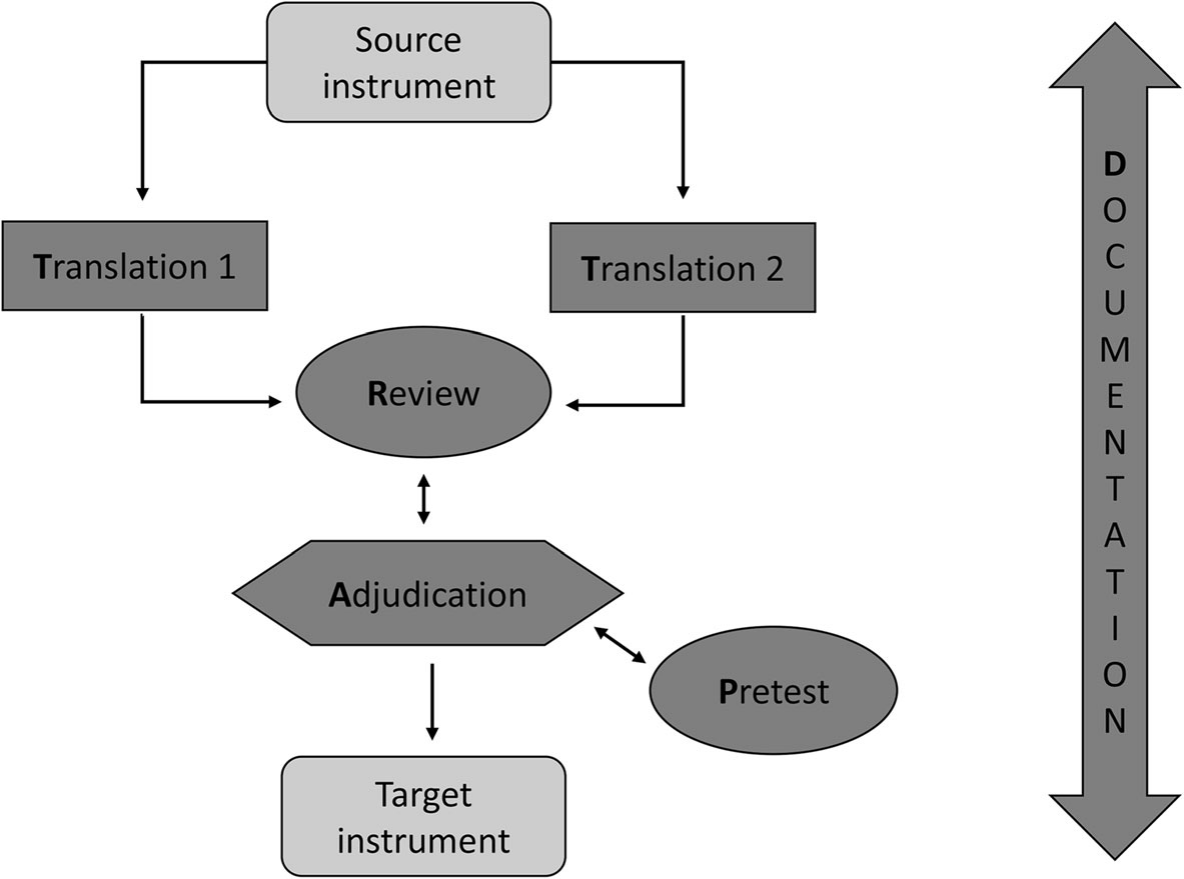
The five steps of the ‘TRAPD’ team translation model

The study was conducted between September 2016 and December 2017. We followed the Translation, Review, Adjudication, Pretesting, and Documentation (TRAPD) team model [15] for cross-cultural translation of questionnaires (see Fig. 1). Two translators, working independently, translated the questionnaire from German to French. A reconciliation meeting took place between the two translators and three reviewers (the main researcher; a senior researcher with survey expertise and a basic knowledge of German, and a bilingual field clinician with a public health background). The panel went through the entire questionnaire, compared and discussed the two versions. The main researcher was responsible for making final adjudication decisions about which translation options to adopt. An online meeting with one of the original authors of the tools and the comparison with an English version drafted by the original development group helped to clarify some ambiguities.

We pretested the version obtained through 8 semi-structured cognitive interviews [16], using a “think-aloud” and on time “verbal probing” technique [17]. We used purposive sampling to select experts representing the target population of the questionnaire, that is to say people with direct responsibilities towards quality of care inside hospitals or their organisational stakeholders, paying attention to heterogeneity in terms of age, gender, education, function and organisational affiliation (see Table 2). Saturation of expert’s reactions to comprehensibility was followed, rather than a predetermined sample size. The interviews were audio-recorded and performed by the main researcher using an interview guide developed for this study (see Additional file 1). Because the primary objective of the interview was to test the quality and appropriateness of the translation with end-users, we explicitly asked the experts to focus first on comprehensibility while browsing through the questionnaire and applying the “think aloud” technique. The interviewer could use emergent probes (reactive and non-standardized) to clarify expert reactions. A secondary objective was to sound out the opinion of the experts on the relevance of the questionnaire in order to tease out possible resistance to its future adoption and to serve its further development. The relevance of each standard, and the overall relevance of the questionnaire, in terms of opportunity for quality improvement, were thus evaluated using anticipated probes (proactive and standardized), supported by a Likert scale (from 0, the least, to 10, the more relevant). The opinion of the expert on the overall applicability of the tool was explored by open questions at the end of the reading. Eight experts, whose characteristics are shown in Table 2, were interviewed for the assessment of the translation. The average length of the interviews was 70 min (ranging from 34 to 107 min).

**Table 2:** Characteristics of the experts interviewed

Age	Mean 50 (standard deviation 17,5)
Gender	2 females, 6 males
Education	● Internal medicine (twice)
	● General practice
	● Nursery
	● Political science (twice, once coupled with interpreting)
	● Law
	● Public health
Function	● Quality project coordinator
	● General practitioner representative inside an hospital
	● Member of ethics committee
	● Quality and safety officer (twice)
	● Quality and safety director
	● Quality project manager
	● Operational manager
Organisational affiliation	● Federal Health Service
	● Belgian Platform for Continuous Improvement of Quality of Care and Patient Safety
	● Health service user’s association
	● Primary Health Care Centre federation
	● Three hospitals whose size ranged from 160 to 1000 beds

A final reviewer meeting validated the main modifications to the questionnaire and the document was then proofread. The documentation of the translation and adaptation process consisted of annotated interview guides and questionnaires, audio-records of the interviews, a table summarizing the main reactions of experts and successive versions of the questionnaires with track-changes and commentaries.

## Results

### Comprehensibility

Overall problems related to comprehension can be described as ambiguity with regard to the chosen word, a need of contextual precision or lack of cultural appropriateness. Non-exhaustive examples are given here for illustrative purpose.

- *Ambiguity in the chosen word* e.g. in the original German version “*der Organisation”* (the organisation) or *“die Einrichtung*” (the facility) were alternatively used and translated in the two first French drafts by either *“l’organisation”* or *“l’établissement”*. The reconciliation meeting noticed the diversity of terms but failed to explicit their specific acceptance. Cognitive interviews revealed a lack of clarity regarding the term “organisation” in French, seen as not evoking something concrete enough per se. This led the main researcher to change the term and to systematize the use of *“l’institution”* when referring to the organisation as understood in its broadest sense or *“l’établissement”*, when understood as an infrastructure, one building or one particular site.
- *Need of contextual precision*: e.g., “*Mittarbeiter/innen”* (staff) was first translated by *“les collaborateurs”* (collaborators). This is another example of ambiguity because it was unclear to experts whether this wording also included occasional and/or non-contractual collaborators with the hospital. So, the term *“le personnel”* (staff) was adopted to restrict the field to the persons working structurally and contractually for the hospital. The experts also expressed the wish to add, where appropriate, *“including physicians”* to include them explicitly as a target group of the item, regarding their somewhat remote position in the organigram of hospitals (in terms of contractual link and/or hierarchical lines) in the Belgian context. Another example of contextual precision brought to the questionnaire was the addition of the remark: *“It* (the organisation) *directs the patient to a mediation service, if appropriate (Law of 22 August 2002 on patients’ rights)”* to item 2.1.5 (addressing the ability for the patient to give feedback and complain about the care that he received). This is intended to reflect a major legal framework, and a standard of care, in the Belgian context*.*
- *Lack of cultural appropriateness*: e.g., experts felt uneasy with wordings possibly related to market rhetoric e.g.: *“Kunde/innen”* (clients) or *“Unternehmen”* (corporate). They preferred the terms *“les patients”* (patients) or *“l’institution”* (the hospital).

At the outset, we also introduced a short glossary stating our choice of the definition of health literacy and to explain clearly the notion of “organisational health literacy”.

### Relevance and applicability of the tool

The main goal of the cognitive interviews was to pre-test the questionnaire in terms of clarity, but, as explained in the method section, cognitive interviews secondarily assessed the relevance and the applicability of the tool. Moreover, the reactions of the eight experts during the “think aloud” step of the process on those different aspects were difficult to dissociate from each other.

**Fig. 2:**
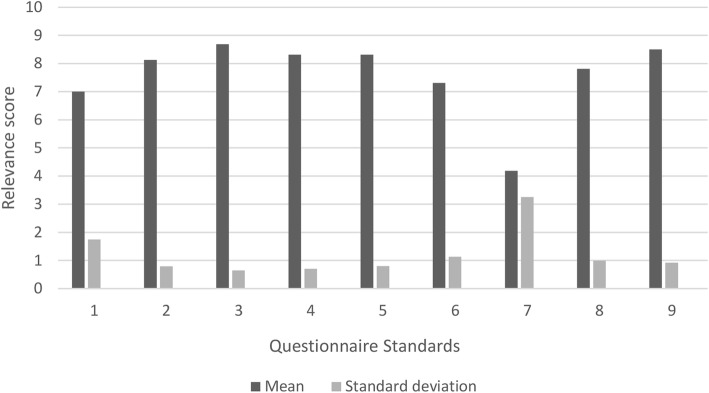
Mean score of relevance for each standard in term of potential to improve the quality of care, as expressed by experts on a Likert scale (from 0 the least to 10 the most relevant)

The experts assessed the French version of the V-HLO tool (V-HLO-Fr) as being globally relevant as in terms of substance. The mean global score of relevance for the V-HLO-Fr tool in terms of opportunity for quality of care improvement as expressed on a Likert scale (from 0 the less to 10 the most relevant) was 7,7 (SD 0,88) (Fig. 2).

Nonetheless, the experts still made some important observations. The most significant of those in our eyes are described below for illustrative purposes (see Additional file 2 for a summary of the reactions of the experts during the cognitive interviews).

- Experts considered standard 7 (the improvement of the health literacy of the staff itself, in terms of self-management with regard to a healthy lifestyle, occupational health and safety risks) as being less relevant for this questionnaire. This affirmation was supported by spontaneous reactions during the “think aloud” step for this standard and confirmed by the mean score obtained for this standard on the Likert scale (see Fig. 2). Quality experts explained that this topic fell under the responsibility of long-established occupational health services and is based on specific legislation. Some of them believed that this standard should therefore not be part of the tool.
  *“You will have to discuss with the union delegation… we are in a completely different world here…we are walking on eggshells*” (expert 5)
- Standard 6 (Improving the health literacy of patients and relatives beyond hospital stay and substandard 8.1 (supporting the continuity and integration of care) were judged by experts expressing a hospital-centric perspective, not paying enough attention to the collaboration with primary health care services, and more broadly with the patient network outside the hospital. They underlined the fact that hospitals should be encouraged to work with this network, in accordance with its capacities, at least from the perspective of transitional care. We thus allowed ourselves to add *“…together with primary care workers and the existing outpatient network”* in the preliminary explanation of standard 6 and *“…together with primary care worker…”* in item 8.1.2 (addressing home return management).
- Some sub-standards and items were considered as falling outside the strict scope of health literacy, sometimes merging with a broader health promotion perspective e.g. substandard 8.2 (encouraging health organisation to contribute to public health in their region).
- Some items, although considered as relevant, were judged too ambitious (or more simply as unrealistic) for the Belgian standard of care, e.g. item 4.1.12 (stating that telephone communication should be available for “most native languages” of patients).
  *“We can always dream”* Expert 5, in reaction to item 4.1.12

The experts adjudged the tool to be applicable to organisational diagnostics while at the same time warning about the risk of discouraging goodwill or potential early adoption by setting objectives that are too high.

*“For some items, the culture is not yet in place or there are not enough resources. If everything is just fallow, it may shock or discourage ”* Expert 2

They also stressed the role of the public authorities to structurally support organisations in their effort to become more health literate.

*“Some tasks, such as creating a folio on colonoscopy for patients, should be performed at a "supra" level, that of public authorities, it would be inefficient to redo it at the level of each hospital”* Expert 4

Facing those reactions, we felt the necessity to write a “preamble to the tool”, to clarify the relationship between health literacy and health promotion and to insist on the necessity of an approach that takes into account existing constraints in any drive to improve quality (see Additional file 3 for an English translation of this preamble).

## Discussion

A valid French version of the original Austrian V-HLO self-assessment questionnaire, the V-HLO-Fr, is now available. This tool could help to raise awareness about organisational health literacy at management level in hospitals. The organisational diagnostic provided by the tool, by highlighting strengths and weaknesses of the organisation, could help to prioritise action. By entrusting the individuals concerned with responsibility for quality, it could create common ground and launch a dynamic around that topic [12]. The V-HLO-Fr could be adopted in other French-speaking contexts providing some light contextual and/or formal amendments to facilitate its appropriation.

Our study has some limitations. The material gathered during the interviews was neither scripted nor systematically coded, because the primary goal was to test the comprehensibility of the draft translation. A more systematic qualitative use of those interviews could still be done within the framework of another publication. The results presented here regarding the relevance and the applicability of the tool are intended to be hypothesis-generating. For example, the reservations of our validation panel in considering standard 7 (improving staff health literacy) as a legitimate and/or reasonable target for the tool may reflect siloed views towards healthcare system functioning, could be a reflection of our relatively narrow user base or indicate delegation of responsibility. Our cognitive interviews were not designed to specifically explore those aspects. In addition, our purposive sampling of experts was partially made by co-optation (that is to say, following the recommendation of previous experts), thus their opinion about the relevance and the applicability of the tool could reflect the position of a particular subgroup, not fully representative of the Belgian French-speaking quality leaders’ hospital landscape.

We could have taken into account more observations from experts in terms of relevance, to adapt the tool more fundamentally to the Belgian context (e.g. going more into details towards better integration between levels of care), but we wanted to maintain its compatibility with the original Austrian version for the sake of a future possible interregional/national comparison. As discussed in the results section, we could still decide to omit standard 7 as a whole for easier implementation into our own context.

A strength of this study is certainly the insight that was gained during the cognitive interviews with regard to the cognitive process of experts when completing the V-HLO-Fr. Those reactions, besides allowing us to improve the translation, made us aware of possible barriers to the future adoption of the tool such as difficulties in considering staff literacy as a legitimate and/or reasonable target for the tool, the fear of overwhelming staff with unrealistic goals, the feeling that some items fell outside the strict scope of health literacy and a sense that there was a lack of attention paid to integration of services with primary care actors. It could also help us to implement the tools more easily in our context (hence, for example, the addition of the preamble to the tool) and, while taking into account the relatively narrow base of our validation panel, drive future improvement of the tool. That work will be carried out in the collegial framework of the working group referenced in the endnote.

The composition of our manager validation panel could also reflect certain inequalities that exist within that group, particularly in terms of gender, and of course does not reflect the diversity of end-beneficiaries (staff and patients). The V-HLO tool is neither the result of a large participatory research, nor intrinsically intended to be implemented in a participatory manner. It is addressed to a narrow user target, in their language, and the goal of our study was not to fundamentally reshape it. This aspect could be considered as an opportunity by remembering that the purpose of self-assessment tools, apart from organisational diagnosis, is to raise awareness and gain support of the management level inside hospital, indispensable in order to allow structural adoption of innovation in such large organisation [12].

But if the tool assumes a top-down approach, it explicitly promotes the participation of staff and patients through some of its items (e.g. by the use of patients or staff surveys, the co-construction of communication tools or the involvement of patients and staff “literacy champions” in training). While keeping in mind that more health literate organisations could, in return, make patient commitment easier at all levels of the healthcare system [18].

The V-HLO tool is essentially focussed on hospitals while most authors of this research are General practitioners. Recurrent testimonies of adverse events obviously connected to health literacy make us, maybe wrongly, to view hospitals as the places where health literacy problems are most frequent and extreme. Our goal, as reflected by some of the slight adaptations brought to the tool, is rather to build common ground that allows health workers at every level to feel responsible for the quality of the whole system [19], in agreement with a broad acceptance of the concept of primary care [20]. Initiatives to develop organisational health literacy tools dedicated to Primary Health Care Centres have also been raised [21] and should be pushed forward.

We are currently running a pilot study in the French-speaking area of Belgium with the V-HLO-Fr tool. Besides performing a cross-sectional organisational health literacy diagnostic within the participating organisations, it will concretely test the applicability and provide a minimal assessment of the impact of the questionnaire in the field. From this perspective, it would be interesting to assess whether this tool can stimulate the emergence of problem-solving spaces between different groups of professionals and a better use of end-user’s experience (staff and patients) to drive continuous improvement.

Finally, as expressed by the experts, Public health authorities should better integrate health literacy into their policies and support organisations in their effort to become more health literate. In that respect, the example of the Scottish action plan for Health Literacy could be inspiring [22].

## Conclusions

We translated and adapted for French the V-HLO self-assessment tool, a questionnaire to help hospitals to identify their strengths and weaknesses in terms of health literacy. If the tool has been judged globally relevant and applicable by experts during the translation process, its implementation is now necessary to assess its feasibility in our contexts, to refine the skills relative to the accompaniment of the intervention within hospitals and to evaluate its real impact in terms of removing barriers created by unnecessary complexities.

## Additional files

Additional file 1: “Interview guide”, English translation of the interview guide used in this study. (PDF 643 kb)

Additional file 2: “Experts reactions”, resume of the reactions of the experts to the V-HLO-Fr during the cognitive interviews. (PDF 851 kb)

Additional file 3: “Preamble”, English translation of the preamble to the V-HLO-Fr questionnaire. (PDF 396 kb)

## Abbreviations

V-HLO: Vienna Health Literate Organisation
V-HLO-fr: French version of the Vienna Health Literate Organisation

## Footnotes

1 See the link below for a short presentation of the working group: https://www.hphnet.org/copy-of-tf-and-wg
