# Supplementary material for: Dealing with health literacy at the organisational level, French translation and adaptation of the Vienna health literate organisation self-assessment tool
Source: BMC Health Serv Res. 2019 Mar 4;19:146. doi: 10.1186/s12913-019-3955-y (PMC6399896; doi:10.1186/s12913-019-3955-y)
Supplement: Supplementary file 1 — “Interview guide”, English translation of the interview guide used in this study. (PDF 643 kb) [file 12913_2019_3955_MOESM1_ESM.pdf]

Validation of the V-HLO-Fr questionnaire

Cognitive interview guide

English translation

**Family name**
**First name**
Education
Function
Age
Gender
If collaborating with an hospital, which one:

Standardised introduction

- Brief reminder of the framework (thesis project and notion of "Organizational Health Literacy") and the objective of the interview (to test a translation with "think aloud and probing" technique)
- Consent for recording?
- Scenario: "You are invited to participate in a group that assesses the quality of care in your hospital using a questionnaire"

1) Clarity ("Comprehensibility")

"Read the questionnaire and think aloud. You do not have to comment on all the items, just do it when a thought comes to you. "

"All your thoughts are interesting, state them all."

"Focus your attention on comprehensibility (the primary goal is to test the translation)."

"Are the phrasing, words and concepts used clear to you? Do they mean specific things that would allow you to answer unambiguously? "

2) Relevance in term of quality improvement

Systematic probing at the end of each standard section and globally at the end of the reading

Standard 1

Comments:

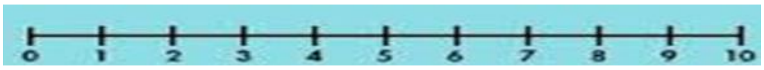

Standard 2

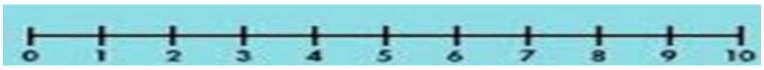

Standard 3

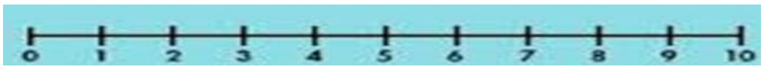

Standard 4

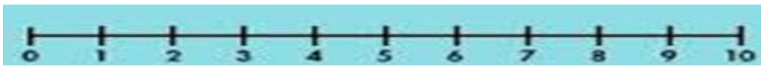

Standard 5

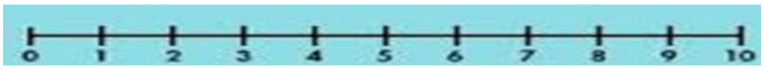

Standard 6

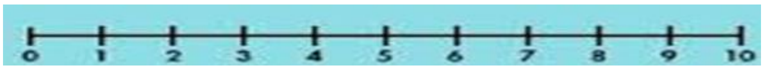

Standard 7

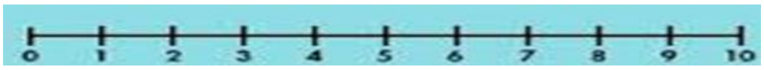

Standard 8

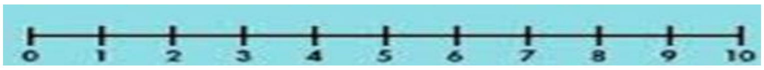

Standard 9

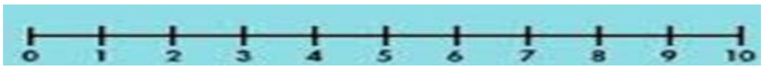

Globally

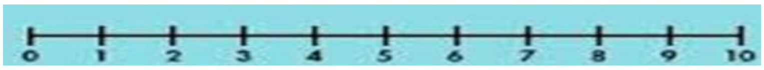

3) Applicability

- How would the tool be applicable to :

  - one-off organisational diagnosis?
  - Benchmarking?
  - other uses?

- Collusion-complementarity with other existing quality approaches?
