# Supplementary material for: Dealing with health literacy at the organisational level, French translation and adaptation of the Vienna health literate organisation self-assessment tool
Source: BMC Health Serv Res. 2019 Mar 4;19:146. doi: 10.1186/s12913-019-3955-y (PMC6399896; doi:10.1186/s12913-019-3955-y)
Supplement: Supplementary file 2 — “Experts reactions”, resume of the reactions of the experts to the V-HLO-Fr during the cognitive interviews. (PDF 851 kb) [file 12913_2019_3955_MOESM2_ESM.pdf]

Resume of the reactions of the experts to the V-HLO-fr[1] during the cognitive interviews

Nota bene, colour code: reactions on clarity in black italic, on relevance in orange, on applicability in red and subsequent modifications in green.

Standards, sub-standards and items	Expert 1	Expert 2	Expert 3	Expert 4	Expert 5	Expert 6	Expert 7	Expert 8
**1**				« …aussi une responsabilité supra institutionnelle (niveau méso) et transversale (enseignement, etc…) »				
**1.1.2**	*Responsabilité* « opérationnelle ? Qui fait quoi ? »							
**1.2.3**	*De la littératie en santé*, « Qu'est-ce qu'on mesure réellement ? » → De la littératie organisationnelle en santé					Idem (et 1.2.2)		

[1] post adjudication meeting draft version

**1.1.5**					*Dans d'autres domaines* → différents domaines « Faire référence à accréditation ? »				
**1.2.4**			*Recueillies qu'une seule fois* « attention à la nécessité d'identito-vigilance » → à des fins administratives		idem				
**1.2.5**						*Compréhensibilité* → clarté			
**1.2.8**	"Systématiquement ? Est-ce bien éthique ? (cf. "screening is not the answer")"								
**1.2.9**	*"Mystery…"* « risque de méfiance » → reformulation et élargissement des moyens		« Bon ! »	Idem que 1					

**2**			*Offres de soins* → prestations de soin		idem			
**2.1**						« Terriblement ambitieux »		
**2.1.3**<br><br>**Attention : exemple de question double**					*Groupe de défense des intérêt* → représentants des patients « sinon sonne trop conflictuel »			
**2.1.5**					→Note : Le cas échéant, elle oriente le patient vers un service de médiation (Loi du 22 août 2002 relative aux droits du patient)	*Intelligibilité* → clarté		
**3**						« deux canaux différents (RH et direction médicale), médecins pourraient ne pas se sentir concernés » → « y compris les	« Pas claire » → reformulation<br><br>*Basic and continuous* → supprimer<br>« pas tous les hôpitaux impliquer dans	

						médecins » en intro	la formation de base et continuée »	
**3.1.1**						« Ajouter descriptif de fonction ? »		
**3.1.6**			« Insérer les patients ? »					
**4**			« Décliner en fonction du type de handicap ? »					
**4.1.8**			idem		« Fournir garantie et/ou explications critiques plutôt que références brutes ? ex. code HON et/ou site gezondheid en wetenschap ? »		idem	
**4.1.11**	idem				« On peut toujours rêver », « coutera trop chère » 4.1.11 et suite 4.1.14		Idem	« Pour les sevices d'urgences oui, mais pas pour l'administratif »
**4.1.12**	idem		idem	« 140 langues à St-Pierre : Assumer le fait et en tirer les	idem	« Dans certains créneaux, selon certaines modalités… »		

				conséquences »				
**4.2.6**	« Pourquoi à cette endroit du questionnaire ? »							
**4.4**	« Aspect « luminosité » manquent »							
**4.4.5**	*Codes couleurs* → élargir à charte graphique				Idem, et « mise en doute pertinence »			
**4.4.7**						« Va vite être le bordel »		« risque de Capharnaüm… »
**4.5.1**		« Plutôt un local pour point d'information oral ? »			« Bof, à supprimer ? »	« Bof »		
**4.5.4**						Difficile		
**5.1.3**	« Tension éthique (confidentialité, droit à l'enfance et mise à mal de la dynamique familiale),							

	renvoie vers 5.4.5 ? »							
**5.1.4**		*De manière détaillée* → de manière personnalisée. « Et pas simplement exhaustive »			Faire référence à la loi sur les droit du patient ?			
					Réceptifs → en état de recevoir de l'information			
**5.2**			« Utiliser matériel des associations »	« Cibler priorité »				
**5.2.9**				« Voir 4.1.8 (redite et idem) »				
**5.4.5**				→ajouter « spécifique »				
**5.4.11**								« Problème juridique avec document de consentement par exemple »
**5.5**		« Définir situations à risque ? »						
**5.5.1**			*Risque* → évènement indésirable		« Ne pas induire culture de peur, l'erreur est			

					opportunité d’apprentissage »				
**5.5.3**					« Les patients peuvent aussi d’initiative notifier »				
**5.5.6**					*Les explications sont données à l’aide des boîtes de médicaments, des piluliers et des comprimés eux-mêmes.* « Controversé, ne pas fermer le champ des possible » Faire référence à transitionnal care et reconciliation ? »				
**5.5.7**			« Sensible, trop détaillé ? »						
**5.5.8**									« Le plus souvent : règle de confinement »
**6**		Idem				« Manque de collaboration avec première ligne et le		« Pourquoi les hôpitaux ? »

						réseau extrahospitalier » → « en collaboration avec la première ligne de soin et le réseau extrahospitalier »		
**6.1.1**			« Cf. 5.1.4 »					
**6.2**			« Quel rapport ? Recentrer sur information ? Qui doit faire cela ? »		« Faire intervenir le réseau ? »			
**7**	« Hors cadre Health Literacy »	idem	Idem « Plutôt gestion du personnelle et culture du risque »		Idem « Attention à délégation syndicale, autre réglementation, médecine du travail, conseiller en prévention etc… », « un autre monde »		Idem « En faire une annexe ? »	« Conseiller en prévention dans chaque hôpitaux »
**8.1**		« Place de la première ligne ? » → 8.1.2 Ajout de	« Hors champs, trop « tuyauterie interne » »			Idem, « hospitalo-centré »	« Serait plutôt 6.3 ! »	

		« les prestataires de première ligne »						
**8.1.7**								« Point de tension déontologie / vie privée) »
**8.1.11**	« Leur donner les moyens de le faire ? »	Idem			« Leur en donner les moyens, les armer »			
**8.2**	« Hors cadre Health Literacy »	idem						
**9.1.5**							« Redondant avec 5 »	

Reactions shared on "central" expressions

- *Client* → patient ; *entreprise* → institution (at large) ou établissement (one particular site)
- *Collaborateurs* → personnels
- *Responsabilité de gestion/de l'entreprise* → responsabilité institutionnelle
