# Supplementary material for: Dealing with health literacy at the organisational level, French translation and adaptation of the Vienna health literate organisation self-assessment tool
Source: BMC Health Serv Res. 2019 Mar 4;19:146. doi: 10.1186/s12913-019-3955-y (PMC6399896; doi:10.1186/s12913-019-3955-y)
Supplement: Supplementary file 3 — “Preamble”, English translation of the preamble to the V-HLO-Fr questionnaire. (PDF 396 kb) [file 12913_2019_3955_MOESM3_ESM.pdf]

Preamble to the V-HLO-fr tool (English translation)

While this questionnaire has the merit of focusing on a somewhat underfunded area of healthcare, it also combines the very limits of the notion of health literacy itself with the dangers inherent in any initiative to improve quality.

We would therefore like to draw attention to the fact that:

- Health promotion cannot be limited to improving health literacy. All the constraints that act as a hindrance to health will not disappear just because information has been simplified and clarified. Nonetheless, this aspect remains important.

-The organizational approach adopted here is aimed at avoiding the trap of highlighting only the responsibility of those individuals who are tasked with taking rational decisions that are informed to a greater or lesser degree. Rather, it aims to view health literacy as a collective responsibility while also taking account of the complexity of healthcare systems and decision-making processes.

-The participation of the users, over and above consultative approaches, must also be seen as a partnership which results in more balanced healthcare, or in participative management methods.

-Finally, the eventual changes suggested by this questionnaire should not overwhelm the staff, respect their indispensable "existing informal knowledge", which is often linked to the specific nature of a given context, as well as their motivation.

Gilles Henrard

General practice department, University of Liège

gilles.henrard@uliege.be
